# A Rare Case of Salmonella Gastroenteritis Presenting as Aspiration Pneumonia With Pleural Empyema in an Immunocompetent Patient

**DOI:** 10.7759/cureus.37192

**Published:** 2023-04-06

**Authors:** Asif Mehmood, Muhammad Fahad Amin

**Affiliations:** 1 Internal Medicine, Lehigh Valley Hospital, Allentown, USA; 2 Medicine, Ghurki Trust Teaching Hospital, Lahore, PAK; 3 Medicine, Jinnah Hospital, Lahore, PAK

**Keywords:** gastrointestinal, pulmonary empyema, gastroenteritis, pneumonia, aspiration

## Abstract

Non-typhoidal salmonella (NTS) can cause infections ranging from self-limited chronic carriers to gastroenteritis, bacteremia, and extraintestinal infections. Pulmonary involvement, particularly empyema, is quite rare and generally found in immunosuppressed individuals. We present a case of salmonellosis in an immunocompetent patient with rare pulmonary complications of empyema. The patient, with no underlying immunocompromised illness, presented with a one-day history of worsening generalized weakness, fever, shortness of breath, and productive cough after having gastroenteritis symptoms of five days duration, which stopped two days prior to admission. On further investigation, imaging revealed right lower lobe pneumonia with empyema. The patient was managed with intravenous antibiotics and chest tube placement with good clinical response. Pleural fluid analysis showed exudative fluid and grew *Salmonella enteritidis* with negative blood and sputum cultures. The patient, in stable condition, was discharged on four weeks of amoxicillin/clavulanic acid after consulting the infectious disease specialist for presumed aspiration pneumonia complicated with empyema in the setting of multiple episodes of vomiting due to gastroenteritis. The lung is an atypical site for salmonellosis. Pulmonary infections in immunocompetent hosts are rare in the medical literature. Early recognition and timely management of pulmonary complications can lead to better outcomes.

## Introduction

Salmonellae are gram-negative rods belonging to the *Enterobacteriaceae* family. It can cause infections ranging from self-limited chronic carrier to gastroenteritis, bacteremia, and extraintestinal infections such as osteomyelitis, meningitis, urinary tract infection (UTI), and endocarditis. The involvement of lungs, particularly empyema, is quite rare [1] and often reported in immunosuppressed patients with comorbidities such as human immunodeficiency virus (HIV), diabetes mellitus, malignancy, long-term use of steroids, or chemotherapy.

This article was previously presented as a meeting abstract/poster presentation at the 2019 American College of Gastroenterology (ACG) meeting on October 26, 2019.

## Case presentation

A 76-year-old male with a past medical history of ischemic cardiomyopathy with 30% ejection fraction (EF) status post-defibrillator, chronic kidney disease stage 3, and previous stroke with residual mild left-sided weakness presented to the emergency room (ER) with a one-day history of worsening generalized weakness, fever, shortness of breath, and productive cough. The patient had been suffering from five days of nausea, multiple episodes of vomiting, and non-bloody diarrhea, which stopped two days prior to admission. Physical examination showed oxygen saturation of 80% on room air with a breathing rate of 24 breaths/minute, fever of 101.1°F, blood pressure of 110/75 mmHg, and pulse rate of 70 beats/minute. He was appearing toxic and acutely ill with lethargy, and his mucus membranes were dry. The belly was soft without tenderness or distention. Breath sounds were decreased at the bases. Laboratory results revealed a white cell count of 14K/uL, blood urea nitrogen (BUN) of 47 mg/dL, and creatinine of 2 with the regular hepatic panel. Chest X-ray revealed small bilateral pleural effusions (the right side greater than the left side) and bibasilar infiltrates/atelectasis.

**Figure 1:**
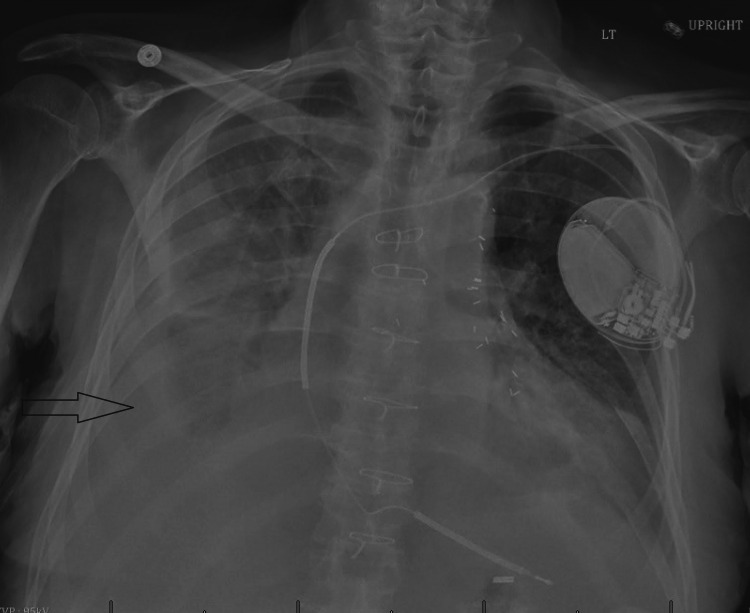
Chest X-ray showing right-sided pleural effusion (arrow)

**Figure 2:**
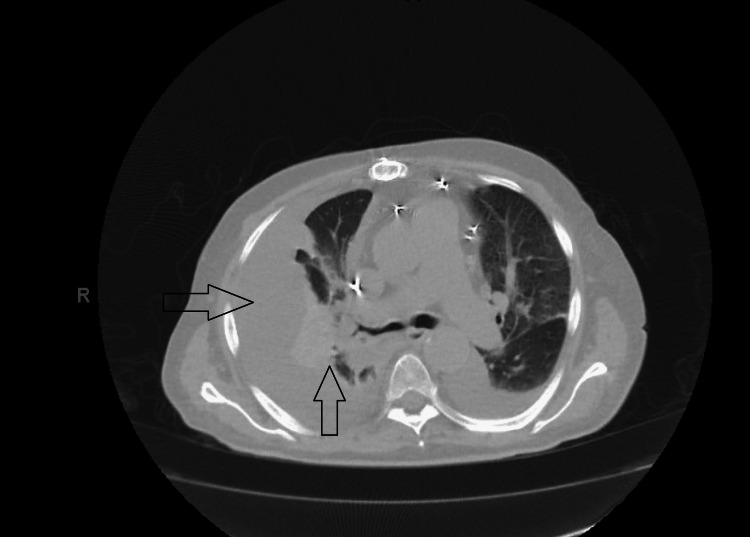
CT scan of the chest showing right lower lobe pneumonia with empyema (arrows) CT: computed tomography

The patient was started on intravenous broad-spectrum antibiotics for suspected right-sided pneumonia. While on antibiotics, the patient continued to have low-grade fevers for the next 1-2 days, along with worsening hypoxia and rapid heart rate greater than 100 beats/minute. The second chest X-ray revealed worsening right pleural effusion (Figure 1). Computed tomography (CT) scan of the chest was obtained, which showed right lower lobe pneumonia with loculated right pleural effusion along with a small left-sided effusion (Figure 2). A chest tube was placed, and antithrombotic therapy was injected to break loculations. Pleural fluid was found exudative and grew *Salmonella enteritidis*. Blood and sputum cultures were negative. In getting further details, recent intake of dairy products was confirmed by the patient, which was likely the source of gastroenteritis. The patient improved clinically, and after consultation with infectious disease, the patient was sent home on four weeks of oral antibiotics (amoxicillin/clavulanic acid) for presumed aspiration pneumonia due to salmonella complicated with empyema.

## Discussion

Salmonella is a facultative intracellular gram-negative bacillus. It is mainly transmitted by the ingestion of contaminated or undercooked food and contact with animal wastes. It is estimated that 1.2 million cases of salmonellosis occurred in the United States, with 23,000 cases of hospitalizations and 450 deaths per annum [2]. Non-typhoidal salmonella (NTS), including *S. enteritidis*, *S. typhimurium*, and *S. heidelberg* usually cause self-limiting gastroenteritis. Among NTS, *S. enteritidis* has been reported as the most frequent cause of lung disease, although geographical distribution plays a role in its prevalence [3,4].

The pathogenesis of salmonella infection is affected by gastric acidity and gastric surgery. Patients using proton pump inhibitors (PPIs) and those with gastric surgery have increased chances of infection. This could be explained by the susceptibility of salmonella to gastric acid [5]. The intensity and risk of translocation are calculated on the basis of the virulence of the isolate and the immunity of the host. In serious cases, patients are at greater risk of death if prompt treatment with antimicrobials is not provided. Elderly, infants, and immunocompromised individuals are at greater risk of severe illness.

In our case, the patient developed gastroenteritis symptoms, including multiple episodes of vomiting followed by pulmonary infection. It was presumed that, most likely, the patient aspirated, resulting in pneumonia, which was complicated further by empyema. No significant underlying etiology was found for salmonella gastroenteritis other than possible exposure to contaminated food as one of his family members also developed similar self-limiting gastrointestinal (GI) tract symptoms.

The lung is an atypical site for salmonellosis, and pulmonary infections in immunocompetent hosts are rare in the medical literature. Only five cases have been reported so far after doing PubMed/MEDLINE search (1966-2019) limited to humans and the English language by using the keywords *Salmonella enteritidis* AND pneumonia AND/OR immunocompetent (Table 1).

**Table 1:** Reported cases of pulmonary infection in immunocompetent hosts IV: intravenous, MRI: magnetic resonance imaging, PPI: proton pump inhibitor, ARDS: acute respiratory distress syndrome

Patients’ age/gender	Risk factors	Presentation	Non-typhoidal salmonella type	Treatment/outcomes	Author name and reference
76 years old/male (our case)	Likely contaminated food, aspiration	Gastroenteritis followed by pneumonia and empyema	S. enteritidis	Treated with chest tube placement, IV levofloxacin, followed by four weeks of per oral amoxicillin/clavulanic acid with full recovery	Asif Mehmood, Muhammad Fahad Amin
24 years old/male	Likely contaminated food	Back pain, fever, rigors, and bloody diarrhea; MRI shows gluteal abscess and sacroiliitis; deteriorated due to bacteremia, pneumonia, and respiratory failure	S. enteritidis	Treated with IV ceftriaxone for 14 days, followed by six weeks of per oral ciprofloxacin with full recovery	Hall, Partridge, Venkatraman, and Wiselka [6]
33 years old/male	Intellectual disability/previously healthy, concern for aspiration	Gastroenteritis and septic shock from pneumonia	S. enteritidis	Empiric treatment with cefepime and levofloxacin for a total of 16 days; discharged to the rehabilitation after tracheostomy	Thompson Bastin, Neville, Parsons, Flannery, Tennant, and Johnson [7]
59 years old/female	Undiagnosed diabetes	Fever, cough, and bacteremia with pneumonia	S. enteritidis	Treated with ciprofloxacin with full recovery	Knight, Knight, and Smith [8]
65 years old/male	Undiagnosed diabetes, undercooked eggs	Nausea, vomiting, diarrhea, abdominal pain, and bacteremia with pneumonia	S. enteritidis	Treated empirically with cefuroxime and erythromycin, followed by oral ciprofloxacin with full recovery	Knight, Knight, and Smith [8]
72 years old/male	Lung cancer with a history of radiation therapy, PPI	Fever, shortness of breath, productive cough, and pneumonia with respiratory failure	S. enteritidis	Treated with sulfamethoxazole/trimethoprim, ceftazidime, and clindamycin; he developed ARDS and expired after five days	Samonis, Maraki, Kouroussis, Mavroudis, and Georgoulias [9]

## Conclusions

Non-typhoidal salmonella causing pneumonia and empyema in immunocompetent patients is very rare but can result in increased morbidity and mortality. We presented the sixth case of its kind, which emphasized the importance of considering non-typhoidal salmonella as a cause of pulmonary infections in appropriate clinical settings. Early recognition and timely management of pulmonary complications can lead to better outcomes.

## References

[1] Jones TF, Ingram LA, Cieslak PR (2008). Salmonellosis outcomes differ substantially by serotype. J Infect Dis.

[2] Crump JA, Sjölund-Karlsson M, Gordon MA, Parry CM (2015). Epidemiology, clinical presentation, laboratory diagnosis, antimicrobial resistance, and antimicrobial management of invasive Salmonella infections. Clin Microbiol Rev.

[3] Dhanoa A, Fatt QK (2009). Non-typhoidal Salmonella bacteraemia: epidemiology, clinical characteristics and its' association with severe immunosuppression. Ann Clin Microbiol Antimicrob.

[4] Zumla A (2010). Mandell, Douglas, and Bennett's principles and practice of infectious diseases. Lancet Infect Dis.

[5] Neal KR, Briji SO, Slack RC, Hawkey CJ, Logan RF (1994). Recent treatment with H2 antagonists and antibiotics and gastric surgery as risk factors for Salmonella infection. BMJ.

[6] Hall RL, Partridge R, Venkatraman N, Wiselka M (2013). Invasive non-typhoidal salmonella infection with multifocal seeding in an immunocompetent host: an emerging disease in the developed world. BMJ Case Rep.

[7] Thompson Bastin ML, Neville NR, Parsons RE, Flannery AH, Tennant SJ, Johnson CA (2016). An unusual case of Salmonella enteritidis causing pneumonia, septic shock and multiple organ failure in an immunocompetent patient. IDCases.

[8] Knight JC, Knight M, Smith MJ (2000). Two cases of pulmonary complications associated with a recently recognised Salmonella enteritidis phage type, 21b, affecting immunocompetent adults. Eur J Clin Microbiol Infect Dis.

[9] Samonis G, Maraki S, Kouroussis C, Mavroudis D, Georgoulias V (2003). Salmonella enterica pneumonia in a patient with lung cancer. J Clin Microbiol.

